# A Systematic Review on Commercially Available and Validated Sensor Technologies for Welfare Assessment of Dairy Cattle

**DOI:** 10.3389/fvets.2021.634338

**Published:** 2021-03-29

**Authors:** Anna H. Stygar, Yaneth Gómez, Greta V. Berteselli, Emanuela Dalla Costa, Elisabetta Canali, Jarkko K. Niemi, Pol Llonch, Matti Pastell

**Affiliations:** ^1^Bioeconomy and Environment, Natural Resources Institute Finland (Luke), Helsinki, Finland; ^2^Department of Animal and Food Science, Universitat Autònoma de Barcelona, Cerdanyola del Vallès, Spain; ^3^Dipartimento di Medicina Veterinaria, Università degli Studi di Milano, Milan, Italy; ^4^Production Systems, Natural Resources Institute Finland (Luke), Helsinki, Finland

**Keywords:** PLF, accelerometer, camera, milk sensor, scale, bolus, dairy cow, calf

## Abstract

In order to base welfare assessment of dairy cattle on real-time measurement, integration of valid and reliable precision livestock farming (PLF) technologies is needed. The aim of this study was to provide a systematic overview of externally validated and commercially available PLF technologies, which could be used for sensor-based welfare assessment in dairy cattle. Following Preferred Reporting Items for Systematic Reviews and Meta-Analyses (PRISMA) guidelines, a systematic literature review was conducted to identify externally validated sensor technologies. Out of 1,111 publications initially extracted from databases, only 42 studies describing 30 tools (including prototypes) met requirements for external validation. Moreover, through market search, 129 different retailed technologies with application for animal-based welfare assessment were identified. In total, only 18 currently retailed sensors have been externally validated (14%). The highest validation rate was found for systems based on accelerometers (30% of tools available on the market have validation records), while the lower rates were obtained for cameras (10%), load cells (8%), miscellaneous milk sensors (8%), and boluses (7%). Validated traits concerned animal activity, feeding and drinking behavior, physical condition, and health of animals. The majority of tools were validated on adult cows. Non-active behavior (lying and standing) and rumination were the most often validated for the high performance. Regarding active behavior (e.g., walking), lower performance of tools was reported. Also, tools used for physical condition (e.g., body condition scoring) and health evaluation (e.g., mastitis detection) were classified in lower performance group. The precision and accuracy of feeding and drinking assessment varied depending on measured trait and used sensor. Regarding relevance for animal-based welfare assessment, several validated technologies had application for good health (e.g., milk quality sensors) and good feeding (e.g., load cells, accelerometers). Accelerometers-based systems have also practical relevance to assess good housing. However, currently available PLF technologies have low potential to assess appropriate behavior of dairy cows. To increase actors' trust toward the PLF technology and prompt sensor-based welfare assessment, validation studies, especially in commercial herds, are needed. Future research should concentrate on developing and validating PLF technologies dedicated to the assessment of appropriate behavior and tools dedicated to monitoring the health and welfare in calves and heifers.

## Introduction

Recently introduced concept of One Welfare recognizes the interconnections among animal welfare, human well-being, and the environment (1). Better understanding of the values of high welfare standards can, among others, support food security, improve productivity, reduce antimicrobial use, and greenhouse gas emission [e.g., (2–4)].

Animal welfare is also a highly interesting topic for European consumers (5, 6). This interest is seen in production statistics and consumer purchases decisions. Consumers are willing to pay a premium price for credence attributes of milk (7, 8), such as organic, environmentally friendly, or high animal welfare (on average 28, 25, and 31% of premium). Moreover, consumers appreciate proactive approach to managing animal health and welfare (9), and there is evidence that the animal-friendly marketing strategies influence the uptake of products (10).

Animal welfare friendly products can be identified through labeling. Most dairy welfare labeling schemes in Europe have requirements concerning resource-based welfare indicators such as space allowance, provision of bedding and enrichments, minimum transportation time, outdoor access, or absence of mutilations (8). Recently, animal-based indicators have gained more attention, especially following the publication of Welfare Quality® (WQ®) protocols and a few labeling schemes highlighting animal-based measures have been introduced during the past years (e.g., AENOR welfare certificate in Spain, Arla one farm milk in Finland, and ClassyFarm in Italy). However, existing animal welfare assessment protocols show some inaccuracies as: (1) they are only applied at group level, (2) are unable to continuously monitor animal welfare, and (3) they rely on human judgments and decisions-making facilitating some degree of subjectivity on the assessment. Moreover, those protocols are not practical for detecting early-warning signals which could result in implementation of preventive measures. Abovementioned limitations could be, at least partially, solved by application of precision livestock farming (PLF) technologies.

Different PLF techniques have been developed for monitoring dairy cattle production. Sudden change in the activity, feeding and drinking, physical condition, and health of animals can be detected by different sensors [e.g., radio-frequency identification (RFID), accelerometers, load cells, and cameras]. Change in behavior or in physical state may indicate problems related to management (e.g., feeding system failure) or disease, as well as can signal specific physiological status such as estrus. PLF technologies can potentially add value to the farm management process by improving data processing, decision making, and implementation of everyday herd management decisions (11). Moreover, PLF technologies could also be applied for monitoring animal welfare [e.g., (12)]. On the other hand, as demonstrated by large-scale studies (13, 14) investment in sensor systems might not necessarily lead to the economic gain for farmers. Therefore, the merits of each sensor system need to be assessed individually and the performance should to be verified before the promise of improved management can be realized.

Research groups and companies around the world has been engaged in developing new PLF sensors, however, not all PLF solutions developed in a lab environment can be successfully implemented as commercial products on dairy farms. The reason can be that some technologies will still be too expensive or will perform better in an experimental setting, where conditions are controlled, and sample size is small, compared to the farming environment. Therefore, for successful assessment of on-farm welfare using PLF technology, it is essential to validate this technology at the commercial level (external validation). Furthermore, applying sensor-based welfare assessment for labeling schemes or welfare support payments should be based on widely available and validated technologies.

The main goal of this review was to assess which welfare aspects of cows', heifers', and calves' husbandry can be addressed by available (and validated) technologies. To reach this goal, commercially available and/or externally validated technologies with potential use for animal-based welfare assessment in dairy herds were first identified. Validated technologies were later grouped according to their performance. Finally, possible gaps between available and validated tools and needs for animal-based welfare assessment were identified based on the principles of the WQ® protocol, including appropriate nutrition, housing, health, and behavior.

## Materials and Methods

### Market Availability Search

A broad market research (using web Google search) on commercially available PLF systems with potential application for animal-based welfare assessment was conducted. This research was done by exploring the assortment of technology providers that cover a wide range of sensors which could provide information on animal base indicators for welfare. The search criteria used included “*dairy cow”* and one of the following terms describing sensors: *(automatic drinker OR automatic waterer), (automatic feeder), (activity sensor OR activity monitor), (RFID), (Global Positioning System OR GPS), (thermal camera), (thermography), (mastitis sensor), (automatic mastitis detection), (somatic cells counter), (milk analyzer), (automatic weigh scale OR automatic weigh), (lameness sensor), (automatic lameness detection), (pressure mat OR force sensor), (body condition score sensor OR automatic body condition score), (body condition camera), (rumen bolus), (milking robot), and (accelerometer)*. Also, search for *calf automatic feeder* was performed. As an example, the following word combinations were used to look for feeding equipment available on the market: “*dairy cow”* plus “*automatic feeder.”* The first five pages (50 hits) from Google search were scanned. Additionally, the availability of sensors was scanned using dedicated on-line marketplace for providers (https://www.agriexpo.online/). Search was performed between March and May 2020. Tools with exclusive use for reproduction (for estrus detection or calving alarms) were excluded from the final list. Information on sensor name, provider name, internet link, sensor type (with attachment position to animal when applicable), aim, and country of origin (headquarters) for 129 technologies are provided in Supplementary Table A1.

### Literature Search and Exclusion Criteria

To explore technology limitation, a systematic literature search based on Preferred Reporting Items for Systematic Reviews and Meta-Analyses (PRISMA) methodology (15) was conducted. Literature search was conducted through Web of Science and Scopus. Altogether, 147 different search terms were used. Each search included terms describing different phases in the production cycle (“*cow” OR “calves” OR “calf” OR “heifer”*) and validation (“test” OR “assess^*^” OR “evaluat^*^” OR “validat^*^”) as well as several exclusion terms: NOT (“*review”* OR “*survey”* OR “*beef”* OR “*sheep”* OR “goat^*^” OR “hors^*^” OR “*buffalo”* OR “steer^*^” OR “*ewe”* OR “*leg calf”* OR “muscle^*^”). Additionally, each search was supplemented with physiological and behavioral term (e.g., feeding behavior), or sensor type (e.g., camera), or the commercial name (e.g., CowView). For physiological and behavioral term as well as sensor types the following terms were used: (“feeding behavior” OR “feeding behavior” AND “monitoring”), (“monitoring feeding”), (“drinking behavior” OR “drinking behavior” AND “monitoring”), (“vocalization”), (“vision”), (“camera”), (“accelerometer^*^”*), (“temperature AND sensor”), (“mastitis AND sensor”), (“image analyses”), (“scale AND body weight”), (“pressure mat”), (“bolus”), (“indoor AND position”), (“in-line”), (“tracking system”), (“RFID”)*, and *(“microphone”)*. The commercial names used in the search are presented in Supplementary Table A1, column A.

The example search looked as follow: (“cow” OR “calves” OR “calf” OR “heifer”) AND (“test” OR “assess^*^” OR “evaluat^*^” OR “validat^*^”*) AND (“camera”)* NOT *(“review”* OR “*survey”* OR “*beef”* OR “*sheep”* OR “goat^*^” OR “hors^*^” OR “*buffalo”* OR “steer^*^” OR “*ewe”* OR “*leg calf”* OR “muscle^*^”*)*.

Only studies in peer-reviewed scientific journals published in English between January 2000 and May 2020 were included to this review. Since this review focuses on dairy production, all validation trials conducted on beef breeds or steers were omitted. Articles were excluded if not dealing with aspects directly related to the welfare of dairy cows (e.g., reproduction related problems such as estrus detection, and environmental aspects such as methane emission, etc.). We further excluded papers with only internal validation, which was defined as validation data set used to assess the performance originating from the same animals or herd/herds as used in the developing of the technology (16).

### Study Classification

This review includes papers presenting the higher standards of objective validation, which is external validation. Based on the approach presented by Altman et al. (16) we have defined two levels of external validation:

1. External self-validation was defined for studies where the system was evaluated using a fully independent data set, that means data was collected from different herds not used for system development. Research was conducted by the same scientist (at least one author involved in developing and validation) or had been validated by at least one author representing a company providing a technology.
2. External independent validation was defined for studies where the system was evaluated using a fully independent data set, which means data was collected from different herds not used for system building. Research was conducted by scientists not involved in technology development.

In order to determine the validation level, both origin of the technology and validation location (herd) needed to be known. Technology was identified through commercial name or based on studies describing building phase (for prototypes). Origin of the validation herd was identified through information on location (country), and type (if a herd was commercial or research). We have assumed that criteria of external validation were fulfilled if commercially available technology was validated in a commercial herd or a research herd (different from the company/developer own research herd). For prototypes, the criteria of external validation were fulfilled only if the scientific paper clearly described where technology was validated, and validation place was different from the herd used for technology building (based on information from scientific publication describing building phase). If both country and herd specifications (commercial or research) could not be identified, then the study was excluded from this review (due to not enough information in materials and methods). However, papers stating that herds used for validation were different than those used for technology development (without mentioning location, for example due to privacy concerns) were included into this review.

### Performance Measures for Validated Trials

In this review, we distinguished regression and classification measures for performance reporting. Regression measures, reflects the agreement between a continuous trait measured by validated technology (predictor) and the golden standard (outcome). For example, the agreement between body weight measured by a conventional scale and partial scale attached to a milk feeder. Regression measures can be presented using any of the following measures including Pearson correlation coefficient (r), Spearman's rank correlation coefficient (rs), coefficient of determination (*R*^2^), mean bias from the Bland–Altman plots (B–A plots), significance tests for intercept and slope of linear regression (I/S), or concordance correlation coefficient (CCC). Classification measures refers to the ability of a technology to predicting categorical outcomes e.g., locomotion score. Classification performance was usually reported using either area under the receiver operating characteristics curve (AUC) or sensitivity (Se) and specificity (Sp) or Cohen's kappa coefficient (κ).

In this review, we have distinguished tools validated for high performance and lower performance. It was assumed that high performance was reached when all indicators defined/selected by authors of studies fulfilled following criteria: r, rs, CCC, Se, and Sp, or AUC was >0.9, *R*^2^ and κ was >0.81, I/S did not differ significantly from 0 or 1, respectively, and B–A plot included zero with the 95% interval of agreement. Criteria for high performance (precision and accuracy) were accepted similarly to those referred by studies assessing technology performance (17–19).

### Assessment of Welfare Relevance

Welfare Quality® is a scientifically rigorous animal welfare assessment protocol (20), which follows four animal welfare principles (good housing, good feeding, good health, and appropriate behavior). WQ® principles were used as a reference to classify indicators measured by technologies. In this review, members of the ClearFarm project with expertise in animal welfare were asked to evaluate the relevance of each indicator measured by the PLF technologies listed in this review for assessing WQ® principles. Possible scores were: “relevant” and “not relevant.” For example, the panel was asked to evaluate whether grazing time is relevant for the principles of good feeding, good housing, good health, and appropriate behavior. Experts votes were categorized based on “relevant” votes, so that all traits with more than 80% votes were grouped in “very relevant” category, traits receiving from above 20% up to 80% votes were in “moderately relevant” category, and all traits with 20% or less votes were in “not relevant” category.

## Results

### Technologies Commercially Available

The full list of commercially available technologies is presented in the Supplementary Table A1. In total, 129 technologies were found from 67 different providers located in 21 countries. The United Kingdom, the Netherlands, and the United States, are the leaders for providing technologies with potential use for animal-based welfare assessment. Technologies were grouped according to the used sensor. Accelerometer-based technologies and load cells were the largest group on the list (37 different products for each group) and constituted 57% of all found tools. Commercially available accelerometers were offered with different animal attachment solutions (collar, leg, ear, and halter), and some companies offered products with more than one attachment option. The collar was the most popular solution (65%, *N* = 24), while leg (30%, *N* = 10), ear (14%, *N* = 6), and halter (3%, *N* = 1) were less frequent. We have identified 14 boluses and 10 products using vision-based monitoring. Regarding milk quality, 25 sensor technologies (19% of a market share) for health monitoring were identified (including 13 milking robots). GPS sensors were used in eight different products offering the possibility to locate animal position. Additionally, two systems using microphone, as well as one mobile app for body condition scoring was identified. All products based on accelerometers offered health alerts. Only one accelerometer-based product was dedicated for calves, the remaining products were advertised for cows or heifers. Systems based on load cells in combination with RFID were most often used for managing and tracking the feeding program of individual animals. Also, few systems were used for body weight monitoring. Boluses were advertised as tools to measure body temperature, pH, and rumen activity as well as for animal identification. Among cameras, seven were dedicated for body temperature monitoring (thermal cameras), two were used for body condition scoring (BCS), and one camera for feeding monitoring.

### Peer-Reviewed Records on Technology Validation

**Figure 1:**
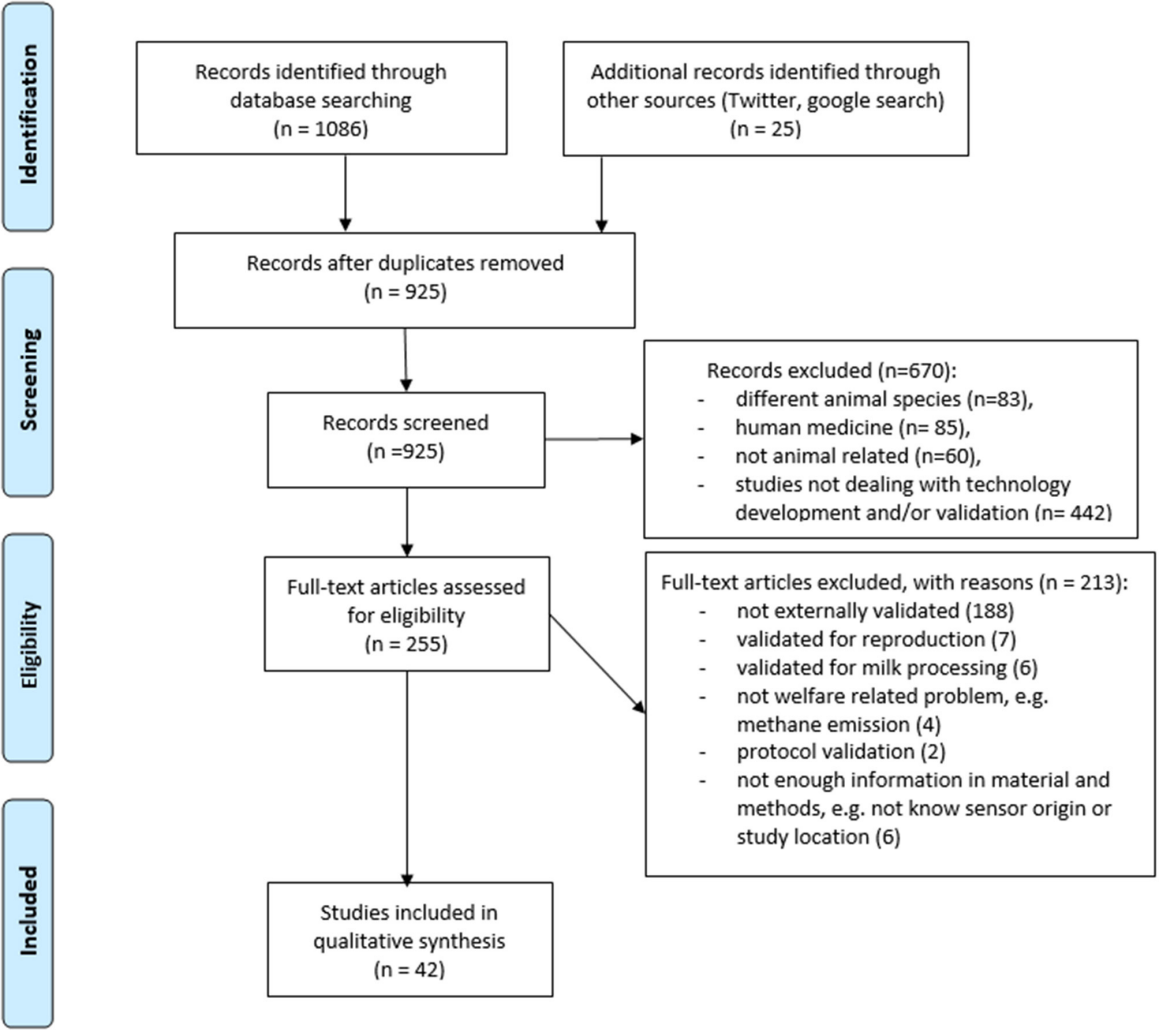
Modified Preferred Reporting Items for Systematic Reviews and Meta-Analyses (PRISMA) flow diagram (15) with the systematic review search strategy and study selection.

The literature search resulted in 1,111 titles, but after duplicate removal and exclusion criteria throughout the review process, 1,069 papers were omitted. A modified PRISMA flow diagram provides information on the number of excluded papers and reason for exclusion (Figure 1). A total of 42 articles satisfied the selection criteria, and 38 publications validated commercially available technologies. Moreover, we have identified four studies on prototype validation (Table 1). Only two papers validated more than one product, however several papers validated more than one indicator measured by the technology. The performance of technologies with accelerometer sensors were the most often assessed (26 technology validation trials). Validation trials for load cells (*N* = 6), bolus, and camera (four trials each), RFID (*N* = 3), microphone and viscosity sensor (two trials each), and conductivity and spectroscopy (1 trial each) were less frequent (Table 1). Regarding accelerometers, the precision and accuracy of products offering different attachments to the animal were assessed in 11 sensors [leg (*N* = 5), collar (*N* = 3), ear (*N* = 2), and halter (*N* = 1)]. The most often validated technology originated from Itin+ Hoch GmbH, Liestal, Switzerland (six trials), Afimilk, Kibbutz Afikim, Israel (five trials), and Agis, Harmelen, the Netherlands (five trials). Information on the study design (herd type, size, and location) for all qualified papers is presented in Supplementary Table A2. In total, 28 studies presented validation trials conducted on research farms. The remaining studies (33%, *N* = 14) were conducted on commercial herds. The sample size used in validation trials varied substantially. In general, the smallest sample size was selected for experiments concerning cannulated cows [below 10 animals for bolus validation, e.g., (52)], while the highest sample size was selected for experiment testing performance of online somatic cell count (SCC) estimation in automatic milking system [above 4,000 milking cows (43)]. When it comes to the geographical location of the herds, most technologies were validated in the United States (11 studies) and Canada (5 studies). The performance of tools was assessed using regression measures (27 papers), classification measures (7 papers), and both measures (8 papers). Most of the reviewed papers were classified as full independent validation, and only 33% (*N* = 14) of reviewed papers were self-validated.

**Table 1:** Summary of externally validated technologies with potential use for dairy welfare assessment.

**Technology name**	**Technology provider**	**No. of validation trials for technology provider**	**Used sensors and attachment position^a^**	**Independent validation^b^**	**Self-validation^c^**
AfiAct Pedometer Plus, AfiTagII, Pedometer Plus	Afimilk, Kibbutz Afikim, Israel (S.A.E.Afikim, Israel)	5	Accelerometer, leg	(21–24)	
AfiLab real-time milk analyzer			Spectroscope	(25)	
CowAlert IceQube, IceTag	IceRobotics Ltd., Edinburgh, Scotland	4	Accelerometer, leg	(21, 26–28)	
Track A Cow	ENGS, Rosh Pina, Israel	1	Accelerometer, leg	(21)	
MooMonitor+	Dairymaster, Tralee, Ireland	2	Accelerometer, collar	(18, 29)	
HerdInsights	Alanya Ltd., Cork, Ireland	1	Accelerometer, collar	(30)	
Hi-Tag	SCR Engineers Ltd., Netanya, Israel (currently Allflex)	2	Microphone, collar	(31, 32)	
RumiWatch	Itin+ Hoch GmbH, Liestal, Switzerland	7	Accelerometer and pressure sensor, halter (29, 33–37), halter and leg (38)	(34, 35)	(29, 33, 36–38)
CowScout Leg	GEA Farm Technologies, Bonen, Germany	1	Accelerometer, leg		(27)
CowManager SensOor	Agis, Harmelen, Netherlands	5	Accelerometer, ear	(21, 39–41)	(17)
The Smartbow	Smartbow GmbH, Jutogasse, Austria	1	Accelerometer, ear	(21)	
Lely activity	Lely, Maassluis, the Netherlands	2	Accelerometer, collar	(42)	
Lely- on-line California mastitis test			Viscosity meter	(43)	
GrowSafe	GrowSafe Systems Ltd., Airdrie, AB, Canada	1	RFID (neck collar), load cell	(44)	
Insentec	Insentec, Marknesse, the Netherlands (now Hokofarm group)	1	RFID (ear), load cell	(45)	
Intergado	Intergado Ltd., Contagem, Minas Gerais, Brazil	2	RFID (ear), load cell		(46, 47)
Body Condition Scoring	DeLaval International AB, Tumba, Sweden	2	Camera	(48)	
Combi			Load cell	(49)	
eCow Farmer bolus	eCow, Dekon, UK	2	Bolus (reticulum)	(50, 51)	
KB 3/04 bolus	Kahne Limited, New Zealand	1	Bolus (rumen sac)	(52)	
Bella Ag Cattle	Bella Ag LLC, USA	1	Bolus (reticulum)	(53)	
Stepmetrix	BouMatic, Madison, USA	1	Load cell	(54)	
Optris	Optris, Berlin, Germany	1	Camera	(55)	
Prototypes—locomotion score	ns	2	Camera		(19, 56)
IMAG model—prototype mastitis detection	ns	1	Conductivity meter, thermometer (milk temperature)		(57)
Prototype-mastitis detection	detection model (prototype) and online cell counter, DeLaval International AB, Tumba, Sweden	1	Viscosity meter		(58)
Prototype-activity	ns	1	Accelerometer		(59)

a *Sensor location is provided only for sensors attached to the animal*.

b *Validated using independent data set (different animals and herd than for technology building) and co-authors were not involved in technology development*.

c *Validated using independent data set (different animals and herd than for technology building) and was developed and validated by at least one the same co-author (based on the authorship of papers) or have been validated by at least one co-author representing a company providing a technology*.

### Validation Rate

According to the obtained results, only 18 commercially available sensors listed in the Supplementary Table A1 have been externally validated (14%). The highest validation rate was found for systems based on accelerometers (30% of tools available on the market have validation records), while the lower rates were obtained for cameras (10%), load cells (8%), miscellaneous milk sensors (8%), and boluses (7%).

### Performance of Technology Validated for Dairy Cows

Table 2 summarizes tools with available validation trials which could have practical application in welfare assessment for dairy cows. Validated animal-based traits concerned animal activity (walking, number of steps, lying, lying and standing, and standing), feeding and drinking behavior (feeding time, presence at feeder, intake, grazing, rumination, drinking duration, presence at a drinker, and water intake) physical condition, and health (locomotion score, BCS, rumen pH, body temperature, health disorder detection, and milk quality).

**Table 2:** Results of validation trials for dairy cows in respect to measured traits.

**Category**	**Measured trait**	**Validation place (farm)**	**Technologies validated for high performance^a^**	**Technologies validated for lower performance^b^**
Activity	Non-active behavior (lying, lying and standing)	Research	AfiAct Pedometer Plus (21), AfiTagII (22), CowAlert IceQube (21), CowManager SensOor (17, 39), Track A Cow (21), RumiWatch (38)	CowManager SensOor (40), Lely activity (42), MooMonitor+ (18), Pedometer Plus (23)
		Commercial	CowScout Leg (27), Ice Tag (26, 27), prototype (59)	
	Standing—identified as a separate behavior	Research	RumiWatch (38)	Lely activity (42)
		Commercial	CowScout Leg (27), Ice Tag (26, 27)	
	Active behavior (walking, no. of steps)	Research		CowManager SensOor (17, 39, 40), IceTag (28), Lely activity (42), RumiWatch (38),
		Commercial		CowScout Leg (27), Ice Tag (26, 27)
Feeding and drinking	Feeding time	Research		CowManager SensOor (17, 21, 39, 40), MooMonitor+ (18), RumiWatch [V0.7.0.0 (33) and V0.7.4.5 34], Track A Cow (21)
		Commercial	RumiWatch V0.7.3.2 (36)	prototype (59), RumiWatch V0.7.2.0 (36)
	Presence at the feeder^c^	Research	GrowSafe (44), Insentec (45), Intergado (46)	
	Feed intake (kg)	Research	Insentec (45), Intergado (46)	
	Grazing time	Research	MooMonitor+ (29), RumiWatch (29, 38)	
	Rumination	Research	CowManager SensOor (17) MooMonitor+ (18, 29), Rumi Watch (29, 33–35, 38), Smartbow (21), Hi-Tag (31)	CowManager SensOor (21, 39, 40), Lely activity (42)
		Commercial	Prototype (59), Rumi Watch (36)	
	Drinking time	Research		Rumi Watch [V0.7.0.0 (33) and V0.7.4.5 (34)]
		Commercial		RumiWatch (36)
	Water intake	Research	Insentec (45)	
	Presence at the drinker	Research	Insentec (45)	
Physical condition and health	Locomotion score	Commercial		Prototype (19), prototype (56), Stepmetrix (54),
	Body condition scoring	Commercial		DeLaval Body condition scoring (48)
	Rumen pH	Research		eCow bolus (50, 51), KB 3/04 bolus (52)
	Body temperature	Research		KB 3/04 bolus (52), OPTRIS (55)
	Health disorder^d^	Commercial		HerdInsight (30)
	Milk quality^e^	Research		AfiLab real-time milk analyzer (25)
	Mastitis detection	Commercial		Lely-on-line California mastitis test (43), prototype -IMAG model (57), prototype (58)

a *All indicators defined/selected in validation trail (by authors of studies) were above high-performance threshold. High precision threshold was reached when Pearson correlation, Spearman's rank correlation, concordance correlation coefficient, sensitivity, specificity, area under the curve (AUC) was higher than 0.9, regression coefficient and Kappa coefficient is higher than 0.81, significance tests for intercept and slope of linear regression did not differ significantly from 0 or 1, respectively, and Bland–Altman plot included zero with the 95% interval of agreement*.

b *Any indicator validated with lower performance (below threshold defined above)*.

c *Animal identification and time*.

d *e.g., mastitis or pneumonia*.

e *Fat, lactose, and protein as indicator for mastitis*.

Non-active behavior (lying, lying and standing, and standing) as well as rumination and feeding time were the most often validated attributes (20, 15, and 11 trials, respectively). There are several different commercially available technologies classified with high performance for non-active behavior (Table 2). For active behavior (walking, number of steps), lower performance of tools was reported. Regarding feeding and drinking, the performance of the tool varied depending on measured traits and used sensor. Feeding time, which was monitored using accelerometer-based sensors, was validated for lower performance. Conversely, presence at the feeder and feed intake (observed in feeding stations) and grazing time (monitored through accelerometer-based sensor) were evaluated for high performance, but only in the research farm conditions. Drinking time was assessed using accelerometer-based tools, and the pressure sensor was evaluated for lower performance. All tools used for physical condition evaluation and health were classified under lower performance. Assessment of locomotion score varied between presented tools [poor (54) or fair classification performance (19, 56)], and in general, none of reviewed technologies was able to outperform the human observer. Regarding BCS, the technology was reliable for dairy cattle with average body condition (scoring between 3.00 and 3.75 on the five-point scale) but did not score accurately for thinner or fatter cows. The only validated study on the accelerometer-based system used for health alarms (30), reported a high number of false positives, but the true health disorders were alerted by the system before the farmer noticed them. Regarding technologies applied for monitoring milk quality and mastitis, real-time milk analyzers agreed moderately with SCC (43), protein, lactose, and fat determined in the laboratory (25), while mastitis detection models have acceptable results for sensitivity, specificity, and error rates (57, 58).

### Performance of Technology Validated for Calves and Heifers

Table 3 summarizes the tools with available validation trials which could have practical application in welfare assessment for young cattle (calves and heifers). Validated traits concerned active behavior (walking), non-active behavior (lying), feeding (time, presence at the feeder, and intake), rumination, drinking (presence at the drinker and intake), body weight, and body temperature. For calves and heifers, rumination and body temperature were the most often validated traits (three and two trials, respectively). Tools measuring active and non-active behavior (lying and walking), feeding and drinking behavior (feed and water intake and presence at the drinker or feeder), and body weight were validated for high performance. Feeding time, rumination, and body temperature were validated for lower performance.

**Table 3:** Results of validation trials for calves and heifers in respect to measured traits.

**Category**	**Measured trait**	**Validation place (farm)**	**Technologies validated for high performance^a^**	**Technologies validated for lower performance^b^**
Activity	Non-active behavior (lying time, lying bouts)	Research	AfiTag II (24)	
	Active behavior (walking, no. of steps)	Research	AfiTag II (24)	
Feeding and drinking	Feeding time	Research		CowManager SensOor (41)
	Presence at the feeder	Commercial	Intergado (47)	
	Feed intake	Commercial	Intergado (47)	
	Rumination	Research		CowManager SensOor (41), Hi-Tag (32), RumiWatch (37),
	Water intake	Commercial	Intergado (47)	
	Presence at the drinker	Commercial	Intergado (47)	
Physical condition	Body weight	Research	Combi DeLaval (49)	
	Body temperature	Research		Bella Ag Cattle (53), OPTRIS (55)

a *All indicators defined/selected in validation trail (by authors of studies) were above high-performance threshold. High precision threshold was reached when Pearson correlation, Spearman's rank correlation, concordance correlation coefficient, sensitivity, specificity, are under the curve, is higher than 0.9, regression coefficient and Kappa coefficient is higher than 0.81, significance tests for intercept and slope of linear regression did not differ significantly from 0 or 1, respectively, and Bland–Altman plot included zero with the 95% interval of agreement*.

b *Any indicator validated with lower precision and or accuracy (threshold defined above)*.

### Experts' Assessment

Answers from animal welfare experts concerning the relevance of the indicator in assessing good feeding, housing, health, and appropriate behavior are summarized in Table 4. For good health, nine traits received “very relevant” evaluation (body temperature, BCS, lameness, mastitis, water consumption, rumination, rumen pH, feed intake, and non-active behavior). Regarding good feeding, seven traits were categorized as “very relevant” (BCS, water consumption, rumination, rumen pH, grazing, feed intake, and feeding time). For good housing evaluation, experts agreed on the usefulness of non-active behavior monitoring. While, for appropriate behavior, only grazing monitoring was evaluated as “very relevant.”

**Table 4:** Indicator evaluation for relevance in assessing good feeding, housing, health, and appropriate behavior^a^.

**Indicator**	**Good feeding**	**Good housing**	**Good health**	**Appropriate behavior**
Body temperature	+−	+−	+	–
Body condition scoring	+	–	+	–
Lameness	–	+−	+	–
Mastitis	–	+−	+	–
Water consumption	+	–	+	+−
Drinking duration	+−	–	+−	+−
Rumination	+	+−	+	+−
Rumen pH	+	–	+	–
Grazing time	+	+−	+−	+
Feeding intake	+	–	+	+−
Feeding time	+	–	+−	+−
Active behavior	–	+−	+−	–
Non-active behavior	–	+	+	+−

a *Symbols +, +−, – refer to “very relevant,” “moderate,” and “not relevant” evaluation, respectively*.

## Discussion

### Retailed and Validated PLF Technologies for Welfare Assessment

The aim of this review was to identify validated and/or commercially available technologies for measuring animal-based welfare indicators in dairy cattle. Currently, farmers can select from at least 129 different sensors to monitor animal-based indicators of health and welfare in dairy production. However, there is still limited information on the performance of these tools. According to our results, only 14% of commercially available sensors have external validation trials available, which may thwart confidence on these technologies.

We identified four potential reasons for such a small number of validation trials: (1) insufficient reporting (2) low scientific interest for validating technology not for research (3) high cost and labor intensity of data collection (4) reluctance to publish negative results.

Regarding reason (1), altogether six studies reporting validation trails were excluded from this review due to insufficient information provided about study design.

Reason (2), there might be lower scientific interest to validate technologies that are not used for research experiments or are not yet integrated as data sources for other systems. For example, for many commercially available sensors based on scales (like individual feed intake measurement), there are no validation trials available. However, the required precision for feeding monitoring tools (as well as the interest in validation) might increase if the data from these tools, as in the example from pig production (60), would be integrated into marketing or health monitoring systems. Furthermore, the validation rate could be increased if technologies, similar as medical industry, receive specific certification [e.g., International Organization for Standardization (ISO) standards]. Currently, devices and systems used for the purposes of official milk recording (e.g., milk meters, samplers, and milk analyzers) need to meet the requirements specified in ISO standards and must be tested to achieve approval from The International Committee for Animal Recording (ICAR) (61). However, the data from the validation process conducted by ICAR are not publicly available. This could also explain a low number of validation records in peer-reviewed literature for milk recording devices and systems.

Reason (3), validation studies can be labor intensive and costly, due to the need to collect the reference data set. For example, accelerometer-based systems are the most widely available and validated among all PLF technologies. But, as demonstrated in this review, the majority of the accelerometer-based validation studies concerned behavioral monitoring and only one validation study for the performance of accelerometer system for health monitoring was found. Validation of health monitoring technology requires obtaining reference data set containing data on veterinary examinations and blood or milk samples to detect among others lameness, mastitis, ketosis, and pneumonia. The substantial costs needed for the reference data set might affect the number of available publications.

Finally, for reason (4), it could be pondered, if the reason behind the relatively small number of validation studies is due to reluctance in publishing negative results. Technology providers are involved in the validation process and altogether, about one-third of all validation studies presented in this review were classified as self-validation. Self-validation could raise the question of conflict of interest in reporting negative results. However, it is impossible to conclude how many of the negative results were never published due to the conflict of interest.

Certainly, technologies which are commercially available may not all have been identified in this study. The search was conducted using internet websites in English, therefore all tools without English marketing material or presented in printed company catalogs were omitted. The biggest producers will have information provided in English, but smaller companies offering products for local markets or startups might not yet have information available for international buyers. Therefore, a constructed list of retailed products is an approximation of the current market. Our goal was not to identify every single technology but to use this list to identify tendencies on the market and set possible market constraints for developing sensor-based welfare assessment. One must also remember that not all validation studies available for a device were reported in this review. We have included only validation studies for attributes related to animal welfare, therefore, some validation studies for performance of estrus detection on accelerometer-based devices [e.g., (62)] or pregnancy detection from in-line analyzer [e.g., (63)] were excluded.

Precision livestock farming uses technology for real-time, continuous monitoring of individual animals and/or groups of animals, which provides an opportunity to improve welfare assessment. Applying sensor-based welfare assessment for labeling scheme or welfare subsidies should be based on widely available technologies. This review shows that reliable technologies for monitoring welfare-related traits exist, however, there are areas concerning sensors and algorithms which require further developments. For example, based on the presented summary, it can be concluded that while recording behavior of farm animals using machine-vision has shown great progress in research (64, 65), it is only entering the commercial market, and external validation will be needed to confirm the performance. Furthermore, according to our results, the performance of existing health and welfare monitoring systems was sporadically tested on young animals (heifers and calves). Validation studies with accelerometers based on collar were rare and only 14% of validated traits for activity monitoring was obtained from the collar devices. On the other hand, this was the most often marketed attachment point for the accelerometer. Therefore, further validation studies for collar-based systems are needed. To successfully assess welfare of young animals, more work on dedicated systems might be required. Further technological and validation gaps regarding assessment of welfare will be discussed according to the principles defined in the WQ® protocol.

### Sensor-Based Welfare Assessment for Dairy Cows and Young Cattle—How Far Are We?

The concept of welfare has multidimensional nature, there is no one indicator that can be used to assess the welfare of an animal, but there are some indicators which are linked to several aspects of welfare. Quite often, welfare assessment is performed using a combination of animal and resource-based indicators (as in WQ® protocol, for instance) and the evaluation is performed by a human observer. Some of the aspects which are evaluated using welfare protocols could be addressed by sensor-based technologies. Below, we will discuss the availability of technologies for the assessment of each welfare principle:

#### Good Feeding

To fulfill the good feeding principle, animals should not suffer from prolonged hunger or thirst. For prolonged hunger, the WQ® protocol adopts animal-based indicator. Regarding thirst criterion, only resource-based indicators are evaluated (20). Therefore, PLF technologies can provide additional single-animal level information for good feeding evaluation.

There were several attributes monitored by PLF technologies (BCS, rumination time, rumen pH, grazing time, feed and water intake, and feeding time), which have high potential for “good feeding” assessment. Some of the attributes (rumination time, rumen pH, grazing time, feed and water intake, and feeding time), when frequently monitored, can be used for designing early warning systems for disease detection and/or feeding system failures [e.g., (66)]. On the other hand, BCS, which assess the proportion of body fat, can have practical application for decision support systems (e.g., predicting the risk of cow developing ketosis or having reproduction problems) (67). The good feeding assessment might be hampered by the commercial availability of technologies. Based on our search, only two providers offered a camera-based sensor used for BCS monitoring. There is also a shortage of tools able to assess grazing time (only two technologies had validation studies for grazing monitoring). Finally, measurements on individual feeding and drinking were performed at feeding stations, mostly used for research (feeding experiments), and due to the high costs of equipment might have little relevance for commercial application. Potentially, systems based on cameras can also have application for feed availability and intake monitoring (68). However, these systems are still in development and only one commercial camera-based system for feed accessibility monitoring was identified. There are several providers of boluses for rumen pH monitoring, but still, relatively little is known on the performance of the detection models (with alarm-based monitoring) for rumen pH monitoring. Additionally, short functional life of the pH boluses [around 40 days due to loss in accuracy of the electrode (69)], does not allow long lasting individual-animal based assessment. Animal presence at the feeding trough or water bin, can be monitored using RFID technologies [e.g., (46)], but available technologies have been tested mostly in experimental farms, and examples with commercial farm validation are rare. Increased competition among cattle at the feed bunk can be currently detected in experimental settings [e.g., (70)] and can indicate shortage of food (decrease feeding time or dry matter intake). However, there is a need for further validation studies on systems based on RFID for detecting food or water shortage at an individual level.

According to our results, good feeding assessment based on animal indicators in commercial settings could be primarily conducted using accelerometer technologies. Accelerometers-based systems are easily available and can assess rumination (with high performance) and feeding time (with lower performance). Moreover, accelerometers together with noseband pressure sensors were used to measure drinking duration [e.g., (36)]. In the future, good feeding assessment could be further improved by integrating information from emerging technologies (such as video-based assessment of BCS).

#### Good Housing

In order to ensure good housing, animals should have thermal and resting comfort as well as enough space to move freely (20). For assessing comfort around resting, the WQ® protocol uses animal-based (e.g. time to needed lie down, animals colliding with housing equipment during lying down, animals lying partially or completely outside the lying area) and management-based indicators (e.g., presence of tethering and access to outdoor loafing area or pasture). Therefore, measuring the activities of animals and the physical state using PLF technologies can provide a more accurate assessment at an individual level. Regarding the evaluation of experts, non-active behavior (lying or standing still) has the highest potential to be used for the assessment of good housing. Allowing dairy cows adequate space and facilities to lie down is considered an important aspect for production as well as animal welfare (71). As recently reviewed, the lying time will depend on individual cow-based factors (reproductive status, age, and milk production), health status (lameness and mastitis), and the comfort of housing facilities (72). For example, pasture-based cows are characterized by longer, undisrupted lying times compared to cows kept in cubicles (73). Lameness can result in longer lying times while mastitis can reduce it (72). For this reason, to avoid confounding factors between animal health and housing conditions, an integration with other data sources, such as milking or breeding records, presence of lameness or mastitis is necessary. Non-active and active behavior as well as grazing time can be assessed using accelerometers. However, performance of technologies varied in different farm conditions. For example, CowManager sensor was evaluated for high (17, 39) and lower (40) performance in measuring lying behavior of cows. High performance was obtained in tie stall and free stall barn and lower performance for grazing cattle. These somehow varying performance results raise the question, if sensor systems should be adjusted (and also validated) for different environmental/ housing conditions. Cleanliness of udder, cleanliness of flank, and cleanliness of upper and lower legs are other animal-based indicators recorded in the WQ® protocol to assess the criterion of comfort around resting and consequently the principle of good housing (20). To the best knowledge of authors, currently there are no available technologies able to assess the cleanliness of animals. However, rapid development in vision-based monitoring for automatic individual identification [e.g., (74)] can prompt the development of algorithms capable of evaluating this welfare aspect. Thermal comfort can be assessed on an individual basis by application of invasive (e.g., boluses) and non-invasive sensors (thermal cameras). Both options are available on the market; however, there is a clear shortage of validation studies for monitoring systems based on those sensors.

#### Good Health

For good health, animals should be free from physical injuries (like lameness and integument alterations) and disease and should not suffer pain induced by inappropriate management or handling (20). As agreed by experts, several traits measured by PLF technologies have a potential application for assessment of good health (body temperature, BCS, lameness, mastitis, water consumption, rumination, rumen pH, feed intake, and non-active behavior). The listed attributes can be categorized as direct or indirect health indicators. Indirect indicators, such as active and non-active behavior, rumen pH, and feeding quantity, on its own does not indicate health status of an animal, but changes in the behavior of animals possibly in combination with other data sources (e.g., lactation status and reproduction) can be processed to obtain early-warning signals for health problems (e.g., lameness, mastitis, and ketosis) and potentially prevent them. Direct welfare indicators, like the number of cows with increased SCC, brings knowledge on health (if an animal is sick or not), and can be useful for operational decisions (e.g., antimicrobial treatment). Injuries, such as lameness, can be detected measuring animal behavior (accelerometers), gait (load cells), posture (cameras), and increased body temperature (thermal camera). Though, the performance of accelerometer-based systems and thermal cameras for lameness detection is unknown (we have not identified external validation studies for lameness detection using those techniques), while the commercial availability and performance of two remaining methods are still low. According to our knowledge, there are no commercially available systems able to detect skin lesions; however, similar to cleanliness evaluation, development in camera-based monitoring systems could in the future allow identification of animals with such problems.

Assessment of good health (and especially presence of diseases) should be based on integrating and analyzing data from different sources. There are commercially available examples of systems using multiple sensors (e.g., milking robots and activity collars) which provide data on milk production, SCC, and animal behavior. System using both an automatic milking system and an activity collar was presented by Elischer et al. (42). However, there are no external validation studies on the performance of these systems for disease detection. There are already examples of flexible models able to handle different sensor or non-sensor data for disease detection [e.g., for mastitis prediction (75)] but the performance of these tools need still to be tested in commercial settings. Even if a technology is not able to provide highly accurate health data on individual level, it could still be useful to estimate herd level prevalence of health problems. Potential integration could concern milk sensor data, accelerometer data, load cells with RFID, boluses (for body temperature and rumen pH), cameras (for body temperature, gait, BCS), and microphones (cough detection).

#### Appropriate Behavior

Appropriate behavior concerns expression of social behavior, expression of other behaviors, good human-animal relationship, and positive emotional state (20). Based on the answers of experts, it can be concluded that PLF technologies currently have a low potential to address appropriate behavior. With the exception of grazing behavior, none of the evaluated attributes was evaluated as “very relevant” by all the experts. However, some of the attributes related to activity as well as feeding and drinking monitoring were evaluated as moderately relevant. From all available technologies, only two tools were tested for evaluation of appropriate behavior (namely, for grazing monitoring). There is, however, a substantial scientific interest in developing research tools aiming to address this welfare principle. For example, accelerometer-based tools were already applied to monitor social behaviors, such as discriminating spontaneous locomotor play (76) and licking/suckling (77) in dairy calves. Also, data from feeding and drinking stations were applied for monitoring social competition (70, 78, 79). In recent years, scientists pointed out the importance of positive emotions as key elements to ensuring good animal welfare (80). In experimental conditions, both ear postures (81) and nasal temperature (82) have been proven to be useful measures of a change in emotional state of cows. For example, the drop in nasal temperatures of cows can be a result of the experience of a positive, low arousal experience. However, further research is needed to design systems able to monitor positive emotional state in commercial settings. Also, there is a technological gap concerning monitoring good human-animal interaction with no retailed technologies intended for this purpose. There is also very scarce information on any experimental techniques for measuring avoidance distance (which is used to assess good human-animal interaction) at the individual level (12). Human-animal relationship could be automatically monitored using 3D cameras, which can capture the distance between a target and camera. However, application of vision technologies requires more research effort.

### Performance Results—Quality and Quantity of Validation Studies

Validation studies are essential for further use of the tool in scientific experiments as well as for welfare labeling or subsidy payments. Therefore, there should be more emphasis on the quantity and quality of conducting and reporting of validation studies for PLF technologies. The results of validation studies are quite often presented as *technical notes* or *short communications* with rather limited space for detail description; however, this does not absolve authors from presenting information necessary for readers to assess the risk of reporting bias. Based on this review, similar suggestions to those presented by Hendriks et al. (83) on how to improve reporting can be made. The location of the trial (commercial vs. experimental herd), criteria for animal selection (e.g., random or based on a stage of lactation), building and management characteristics (e.g., floor type and grazing), and feeding system should be always reported, since results obtained in the different production settings might not be comparable. Furthermore, the validated tool, especially if not commercially available, should be described in enough detail for correct technology identification. Also, when possible, the software version should be reported.

In this review, we have grouped tools based on reported performance measures. Threshold for high performance was selected based on available literature and represents very good agreement. Here, it should be noted that tools which did not fulfill high performance criteria still have practical relevance. For example, online California Mastitis Test performed by milking robots, agreed only moderately with laboratory measurements on SCC. Even though this data was not very precise, nevertheless can be very useful for on-farm decision making, due to the high sampling frequency (43). Therefore, the practical relevance of tools need to be assessed based on their objectives (84) and judged one by one. The results from a single validation trial are not yet conclusive regarding the tool performance. Ideally, tools should be validated in different production conditions (e.g., different countries and housing). According to our results, some of the traits were validated multiple times, by different research groups around the world. And as expected, some presented performance results were inconsistent (as in the example of the CowManager sensor evaluated in different housing conditions). Also, as seen from the example of Supplementary Table A2, authors varied in reported statistics. There seems to be no clear guideline on sufficient level of information regarding performance which should be reported. For example, for classification models, reports should not be based on presenting only sensitivity and specificity of the model without information on selected thresholds for detection. Instead, the performance of the classification model should be preferably presented in receiver operating characteristic curve, which is the overall performance indicator (85). Regarding regression models, the adequate statistical tests are presented for example in Tedeschi (84). Including only Pearson correlation coefficient allows assessing precision of the tool, while nothing is known about its inaccuracy (the systematic deviation from the truth). Testing for tool performance is especially important for technologies from which data will be post-processed and used for building further algorithms. In this review, we have not removed or distinguished in result table studies which provided somehow limited information on the tool performance (for example, only results on Pearson correlation). It could be possible to set additional exclusion criteria for papers selection; however, one must remember that even in the limited form, these studies provide some partial information about the validity.

### Application of Sensor-Based Technology for Welfare Assessment on Farms and Beyond

The primary goal of a sensor system is to improve animal management. Sensor systems provide information for decision making which may, among others, influence farm profitability and animal health and welfare as well as have environmental impact (11). However, potential application of sensor systems can go beyond a single farm level. There are studies demonstrating that data routinely recorded from milking robots provide information which can assist in genetic evaluation [e.g., (86)]. Moreover, production data could be utilized for designing health surveillance systems. For example, an attempt was made to use milk yield data to detect outbreaks of Bluetongue and Schmallenberg viruses (87). PLF technologies may provide evidence-based approach to the monitoring and surveillance of animal welfare not only at the farm but also during transport or at slaughter (88). Already now, in some countries, there are suggestions to base the certification system of livestock farming on real-time measurements and using animal behavior as a criterion for quality labeling (89). This kind of policy could increase transparency of the sector and could result in a wider selection of welfare friendly products. As demonstrated in a previous review, data routinely collected on the farm (e.g., on milk yield, culling, and reproduction) and available in national data base, were associated with dairy cow welfare (90). Also, meat inspection data can have practical application for welfare assessment (91). This review demonstrates that data collected during on-farm monitoring has high potential to assess different aspects of dairy cow welfare, and that currently available technologies can provide animal-based welfare information. However, for the data to be fully utilized for this purpose, there is a need to develop new methodologies for data integration and processing. Data collected from various automatic recording technologies need to be processed and integrated into a single outcome of animal welfare (which is easy to understand by the consumer). This challenging task will be considered by the ClearFarm project, which aims to develop a platform to control animal welfare in pig and dairy farming. The integration of technologies for welfare, health surveillance, or breeding evaluation will require access to a vast amount of PLF data from different devices and different users. The utilization of these data requires that data ownership rights, privacy, and confidentiality issues are resolved and agreed between the parties involved. For example, for the EU markets, non-binding guidelines on data sharing from PLF technologies are available (92) and cover, among others, ownership, access, control, and privacy. However, according to the recent review on digital agriculture, the area of data ownership regulations could receive more attention (93). Another challenge concerns data storage capacity and strong computational power. However, there are already efforts to design a set of industrial, large-scale high-performance computing solutions to support the processing of very large PLF data sets from different users (94).

## Data Availability Statement

The original contributions presented in the study are included in the article/Supplementary Material, further inquiries can be directed to the corresponding author/s.

## Author Contributions

MP, JN, AS, EC, and PL conceived the presented idea. AS defined literature search. AS, GB, and EDC carried out literature search. YG defined and performed company search. AS wrote the paper with input from all the co-authors. All authors contributed to the article and approved the submitted version.

## Conflict of Interest

The authors declare that the research was conducted in the absence of any commercial or financial relationships that could be construed as a potential conflict of interest.
